# Cajal and Pavlov: a comparison between two central neuroscientific schools of the twentieth century

**DOI:** 10.3389/fnana.2014.00122

**Published:** 2014-11-21

**Authors:** Carlos A. Blanco

**Affiliations:** Facultad de Ciencias Humanas y Sociales, Universidad Pontificia de ComillasMadrid, Spain

**Keywords:** Cajal, Pavlov, methodology, neuroscientific schools, Spanish neuroscience

Santiago Ramón y Cajal (1852–1934) and Ivan Pavlov (1849–1936) share some important features in common: both of them made outstanding contributions to the study of the brain; the two of them came from peripheral countries which had remained in deep isolation from the principal centers of scientific progress in the nineteenth century (United Kingdom, France, and Germany); both of them achieved great recognition in their lifetime. Pavlov received the Nobel Prize in physiology or medicine in 1904; Cajal won it in 1906, together with the Italian physician Camillo Golgi (1843–1926). Moreover, Cajal and Pavlov established well-known neurological schools in their respective countries, some of whose disciples became prominent figures in the fields of anatomy and physiology.

Cajal's discovery of the individuality of nerve cells opened the way for some of the greatest neuroscientific developments of the twentieth century. He refined Golgi's silver staining technique and he offered a detailed description of the anatomy of the nervous system, culminating in his monumental *Textura del Sistema Nervioso del Hombre y de los Vertebrados* (1904–1906), perhaps “the most original work ever written in Neurology” (De Castro, 1981, pp. 31–32).

Initially interested in the study of digestion, Pavlov revolutionized the disciplines of psychology, and physiology by elucidating the nature of conditioned reflexes after a series of famous experiments with dogs. He and his disciples discovered the role of the brain in salivary and gastric secretion (Pavlov, 1902). His influence on our understanding of some forms of learning has been enormous (Todes, 2000, pp. 97–104),especially through key figures of the behaviorist school like John Watson (1878–1958).

Pavlov headed the department of physiology at the Imperial Institute for Experimental Medicine in St. Petersburg from 1890 until his death in 1936: around 45 years of leadership over numerous prominent disciples. Topics like the innervation of gastric glands, the physiology of pancreas and conditioned reflexes were fruitfully examined. His fame attracted relevant international professors and researchers, eager to work with the renowned Russian scientist. For example, in 1902 professors Konheim (Heidelberg University) and Chermak (University of Halle) carried their research under the direction of Pavlov. Russian disciples of Pavlov like Krasnogorsky and Nikiforovsky applied the study of conditioned reflexes to pharmacology. From 1891 to 1917, when the Russian Revolution took place, “more than 110 persons worked here during different periods of time under the direction of Pavlov” (Klimenko and Golikov, 2003, pp. 115).

The so-called “Spanish Neurological School,” tragically affected by the 1936–1939 Civil War, was founded by Cajal. One of its principal precursors was Luis Simarro (1851–1921), and it had prominent names like Fernando de Castro (1896–1960), who made important contributions to the structure and function of sympathetic ganglia, the study of baroreceptors and the organization of synaptic complexes; Nicolás Achúcarro (1880–1918), known for his research on the macroglia and the architectonics of neuroglia in cerebral cortex; Pío del Río-Hortega (1882–1945), who discovered microglia and created an important histological method; and Jorge Francisco Tello (1880–1938), who studied the process of degeneracy and regeneration of nervous endings and contributed to our knowledge about the development of the nervous system (Gallego, 1983). The official founding date of this school was 1902, when an Institute for Biological Research was created so that Cajal, who had been recently awarded the Moscow Prize at the International Congress of Medicine that had taken place in Paris in 1900, could continue his work. Tello and Domingo Sánchez (a future leading researcher on the invertebrate nervous system) were among the first to participate in the new center and collaborate with Cajal. Achúcarro joint the Institute in 1911, and in the following years an amazing amount of neuroscientific achievements flourished (De Castro, 1981, pp. 57–61).

However, the working methodologies of both schools showed significant differences. The two schools achieved an almost unequaled degree of productivity in the first decades of the twentieth century, but whereas Pavlov's school preferred a more hierarchical organization, Cajal tended to grant more freedom to his students and collaborators (De Castro, 1981, pp. 51). According to Fernando de Castro, Cajal offered higher levels of independence and free intellectual movement to those who worked with him. In Pavlov's laboratory, every scientist seemed to fulfill an “organic,” and impersonal function, in which the footprint of the individual disciple became minimized. Pavlov exerted an almost complete control over the work of his collaborators. In the prolog to his book *The Work of the Digestive Glands* (1902), an English edition in which he summarized some of his principal discoveries, he remarks that the meaning of a certain experiment must be understood from the viewpoint of the “Laboratory,” not of the individual researcher. De Castro names this methodology “direct” collaboration (De Castro, 1981, pp. 49), and he opposes it to the “indirect” way privileged by Cajal. In Cajal's school, the disciple could choose the research topic that was closer to his scientific interests. The master was regarded as an example to imitate and a source of advice rather than a true hierarchical authority to which he should report all his ideas, developments and findings.

The outstanding productivity of both schools indicates that the paths toward great scientific discoveries follow no general rule. Two schools guided by two different methodological principles managed to make some key contributions to our understanding of the nervous system. However, the greater freedom to pursue individual research that Cajal granted to his disciples may explain why the Spanish Neurological School managed to produce relevant figures in the field even when the activity of their master had declined or had disappeared.

The two schools also share a tragic destiny: the Civil War (1936–1939), and the diaspora of researchers to countries like Argentina and Mexico, meant the abrupt ending of the most glorious period of neuroscientific research in the history of Spain; after the 1917 Revolution, the international isolation suffered by many Russian scientists deprived Pavlov's school from its past splendor.

## Conflict of interest statement

The author declares that the research was conducted in the absence of any commercial or financial relationships that could be construed as a potential conflict of interest.
